# ‘It only works if we coordinate our work, that’s what we need’: a relational qualitative network analysis of exercise therapy in oncology care in Germany

**DOI:** 10.1186/s12885-025-15326-y

**Published:** 2025-11-29

**Authors:** Saskia Blütgen, Anna-Lisa Tigges, Anna Zinkevich, Katja Krug, Michel Wensing, Katharina Graf, Joachim Wiskemann, Lena Ansmann

**Affiliations:** 1 Faculty of Medicine and University Hospital Cologne, Institute of Medical Sociology, Health Services Research and Rehabilitation Science (IMVR), Chair of Medical Sociology, University of Cologne, Cologne, Germany; 2 Department of Health Services Research, School of Medicine and Health Sciences, Carl von Ossietzky University of Oldenburg, Oldenburg, Germany; 3 Department of General Practice and Health Services Research, Heidelberg University and Heidelberg University Hospital, Heidelberg, Germany; 4 Department of Oncology and Haematology, Krankenhaus Nordwest and University Cancer Centre (UCT), Frankfurt am Main, Germany; 5 Department Medical Oncology, University Hospital and National Centre for Tumour Diseases Heidelberg, DKFZ and University Medical Centre Heidelberg, Heidelberg, Germany

**Keywords:** Cancer, Social networks, Relationships, Exercise therapy, Oncology

## Abstract

**Background:**

Despite robust evidence on the effectiveness of exercise therapy care as a supportive strategy in cancer care, access for cancer patients remains limited across Germany. This is due to a lack of renumeration opportunities, insufficient knowledge, and the absence of integrated provider networks. Existing care services have mainly arisen from individual initiatives and the unwavering commitment of sports science pioneers, who devoted their time, energy, and expertise to developing better methods of care. This study analyses the informal provider networks in the emerging field of exercise therapy care and research, focusing on recurring patterns of interaction between exercise therapy providers and medical professionals that enable patient access to exercise therapy care.

**Methods:**

This study employed a combination of qualitative network analysis and contextual analysis. A total of *n* = 31 qualitative interviews were carried out with representatives from the provider networks of exercise therapy care within seven comprehensive cancer centres (CCCs) to explore current patterns of care. The interview data were analysed using qualitative content analysis following Kuckartz, supported by MAXQDA software. Contextual analysis was supported by a survey of key informants (KIs) from exercise therapy care units in CCCs (*n* = 7) to collect data on patient flow and CCC characteristics.

**Results:**

We identified seven recurring relational patterns of operation that facilitate patient access to exercise therapy care within care networks. These patterns include mutual recognition from previous projects and shared facilities, improved accessibility to exercise therapy care options, the development of reciprocity between supportive services, raising patient awareness and sensibilisation through existing patient navigators, planning for patient needs via structured screenings for supportive services, collaboration with medical care, and the sharing of knowledge through informational events for healthcare providers.

**Conclusion:**

Integrated provider networks of exercise therapy care have developed informally, resulting in structural fragility and limited robustness. As long as the effectiveness of exercise therapy care remains research-driven rather than embedded in standard care pathways, these networks remain vulnerable. Establishing formal policies and stable reimbursement mechanisms is essential to institutionalising network ties; strengthening social capital; and ensuring resilient, sustainable access to exercise therapy for all cancer patients.

**Trial registration:**

ClinicalTrials.gov (Clinical Trials Identifier NCT06185777), December 29, 2023.

**Supplementary Information:**

The online version contains supplementary material available at 10.1186/s12885-025-15326-y.

## Background

### Exercise therapy care for cancer patients

In Germany, 504,166 people were diagnosed with cancer in 2022, and 230,258 died from the disease [1]. As a result, cancer ranks as the second leading cause of death in the country. However, cancer survival rates have been increasing each year. Between 2017 and 2018, 51% of men and 59% of women survived cancer for at least five years after diagnosis [1]. Upon diagnosis, patients typically undergo various treatments such as medication and surgery. In addition to standard medical care, supportive services may be available to address patients’ psychosocial and physical needs as well as their overall well-being. These services typically include psycho-oncology, nutritional therapy, social services, and exercise therapy care [2].

In recent decades, cancer research has focused largely on medical treatments such as surgery and chemotherapy and their effectiveness and tolerability [2]. While advances in cancer treatment have significantly improved survival rates and reduced side-effects, comparatively less attention has been given to exercise therapy care as a supportive strategy for cancer care [3]. We define exercise therapy care as the entire process of patient care that includes (1) providing information or counselling about exercise therapy options and programmes as well as (2) the actual provision of exercise therapy through physical activity, either indoors or outdoors, with or without training equipment.

A growing body of evidence highlights the importance of physical activity and exercise in oncology. Exercise therapy care (e.g., exercise therapy, physical activity) has been shown to improve patient outcomes, manage treatment-related side effects, and contribute to a more holistic approach in cancer care [3–5]. However, in Germany, the implementation of exercise therapy care in cancer care remains limited due to insufficient healthcare structures, lack of patient access and knowledge, and insufficient reimbursement [6, 7]. Despite the extensive body of research evidence supporting exercise therapy as an effective intervention in supportive cancer care, the provision of exercise therapy remains limited. Thus, exercise therapy serves as a prime example of how evidence alone is insufficient to guarantee patient access patients [8, 9]. Consequently, an implementation gap persists. In this paper, we therefore examine the current provider networks of seven oncology exercise therapy care units in Germany.

### Social network analysis in oncology exercise care

Social network analysis serves as a methodological tool that integrates theoretical approaches to social behaviour from both macro- and micro-level perspectives [10]. Granovetter [10] emphasised that the ‘analysis of processes in interpersonal networks provides the most fruitful micro-macro bridge’ (p. 1360). Qualitative social network analysis offers valuable insights into how relationships within a network are formed, maintained, and evolve over time [11]. These insights are crucial for designing and implementing effective healthcare strategies that address the complexities of interpersonal and organisational dynamics [12, 13]. Qualitative social network approaches are particularly relevant in healthcare organisations, which are characterised by their innovativeness, multidisciplinary nature, and the complex interactions between several professions [3, 14]. The goal of some networks among various stakeholders in healthcare systems is to dismantle barriers between different units within healthcare organisations (e.g., hospital-based primary care and supportive services such as exercise therapy care and psycho-oncology) and to promote more accessible and comprehensive care [15].

**Fig. 1:**
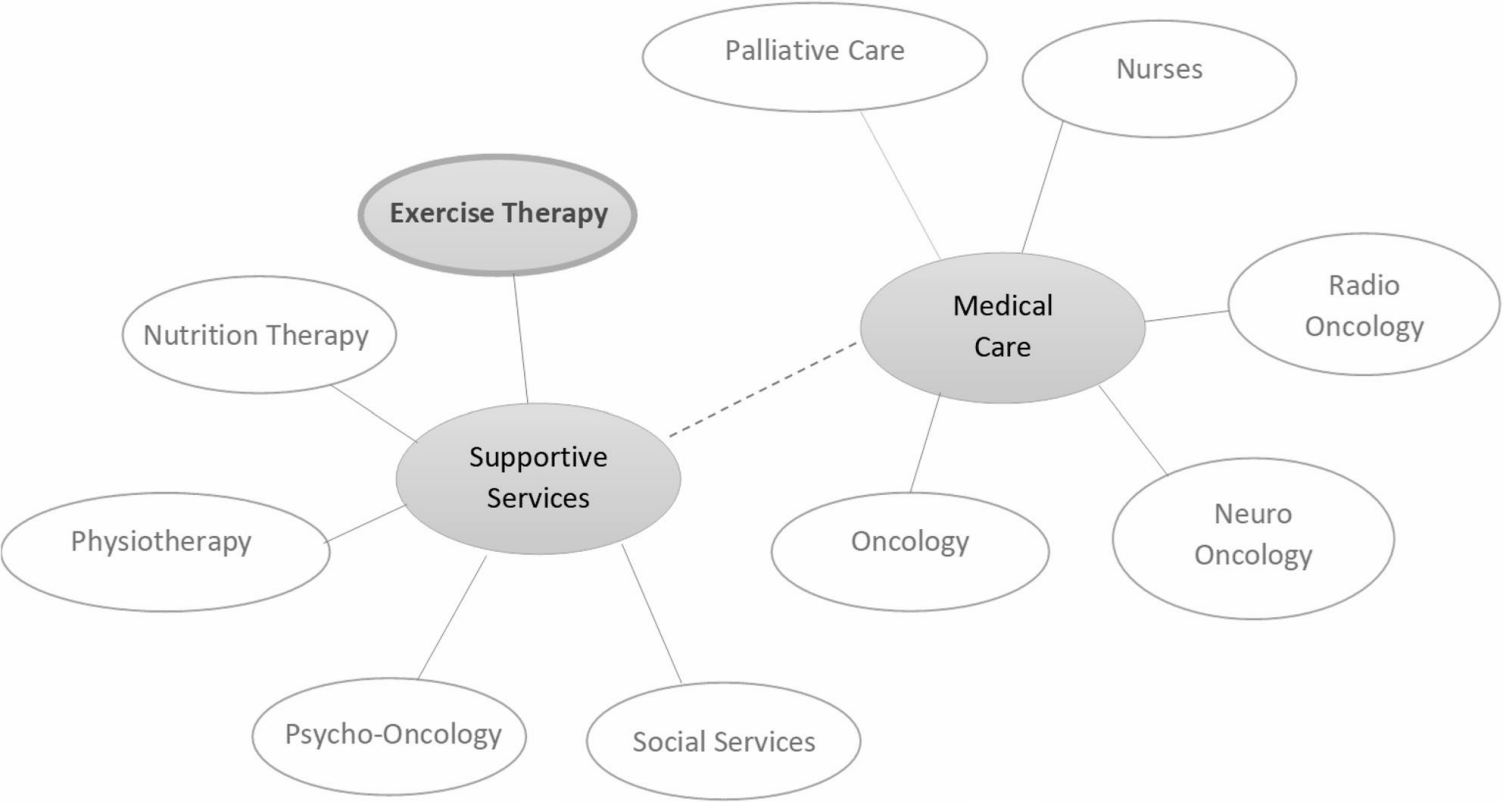
Potentially relevant actors for patient access to exercise therapy care

Social networks in exercise therapy care encompass a diverse range of actors within a healthcare organisation, including oncologists, nurses, and supportive services (see Fig. 1). However, ensuring access to exercise therapy care also requires broader collaboration with external actors, such as community organisations, rehabilitation centres, physiotherapy providers, general practitioners, family members, friends, and policymakers [6]. Networks in exercise therapy care are newly developed and constitute a growing field of care [16]. Thus, we view them largely as informally established structures that emerge around individual initiative and social capital. This view is informed by the relative novelty of the field, the absence of established institutional care pathways, and the limited integration of exercise therapy into standard oncology care.

### Social capital in exercise therapy care networks

The definition of social capital varies depending on the theoretical perspective. Bourdieu [17], one of the pioneers in this field, described social capital as a tool of power reproduced through exclusive networks and that reinforces social hierarchies. In contrast, Putnam [18] viewed social capital as beneficial for the common good, fostering cohesion through trust and cooperation. While Bourdieu emphasised social inequality, Putnam highlighted societal benefits. Lin [19], however, offered a slightly different perspective by focussing on individual gains within social networks. Lin [19] defined social capital in a network context as a resource that individuals can leverage through strategic network positions for personal advantage. Within social networks, actors possess different resources, some belonging to individuals and others primarily held by other actors, accessible only through social relationships. Lin’s [19] conceptualisation is particularly compelling because it underscores the role of social networks in creating and mobilising resources embedded within the networks. This network-centric perspective highlights the value of social capital in facilitating access to information and support [19].

As there are currently no comprehensive structures for exercise therapy care in cancer care, patient access remains limited, creating a gap between research and practice. This gap requires analysing the informally built social networks of exercise therapy care and identifying their operational principles as common relational patterns that strengthen social capital.

## Aim and research question

In this paper, we examine social networks through a qualitative network analysis embedded in a contextual analysis [20]. These networks are analysed in terms of their operational principles, which are defined as recurring patterns of relational interaction between exercise therapy care providers and medical healthcare professionals that enable patient access to exercise therapy care. Accordingly, the research question is as follows: What are the operational principles of social networks in exercise oncology care?

## Methods

This study is part of a larger project: Multi-professional care pathways and networks for the promotion of needs-oriented, resident-oriented exercise therapy care for oncology patients (MOVE-ONKO), which is a multi-professional care pathway and network for the promotion of needs-oriented, resident-oriented exercise therapy for oncological patients. The MOVE-ONKO project includes a complex intervention that aims to establish and pilot a comprehensive care network for multi-disciplinary exercise promotion and therapy. After a status-quo evaluation of the current care networks in the seven CCCs a structured care pathway will be implemented across seven comprehensive cancer centres (CCCs; for further details, see the study protocol [21]). CCCs are leading oncology centres nationwide and are dedicated to reducing cancer incidence through excellence in clinical care, research, and education [22]. The MOVE-ONKO project includes a mixed-methods evaluation study structured into three phases: before implementation (phase 1, the focus of this paper) during implementation in CCCs (phase 2), and during a roll-out of the pathway to additional cancer centres (phase 3).

### Research design

Given the exploratory nature of the research subject and the recent growth in supportive services in oncology healthcare, qualitative methods are particularly well suited to investigating the common pattern of social networks in exercise oncology care. The Consolidated Criteria for Reporting Qualitative Research (COREQ) checklist was used as a reporting guideline [23] (see Supplement I). To assess the care situation before implementation, a qualitative network analysis with qualitative semi-structured interviews embedded in a contextual analysis in phase 1 was used [20, 24]. The contextual analysis considers the broader social, structural, and environmental factors that influence interactions within a given setting [20, 24]. By examining provider networks within their specific context, this combined approach helps to shed light on the existing care settings and networks in the seven CCCs. Implementing comprehensive and structured care pathways for exercise therapy care in cancer care requires a broader perspective; to this end, the context analysis based on the context and implementation of complex interventions (CICI) framework is applied alongside Lin’s social capital approach. According to Lin [19], networks are structures through which resources are used to achieve providers’ individual goals while also contributing to a wider objective, in this case, providing exercise therapy care to all patients. Actors within the network must collaborate across all relevant disciplines and care boundaries to achieve this goal. The following section explores which network resources are already in place. Therefore, the interviews focused on provider networks, and the CICI framework was used to analyse the preconditions based on the dimensions of setting and context of exercise therapy care networks.

Key informants (KIs), namely the leaders of the exercise therapy care units with extensive knowledge of exercise therapy care, were interviewed to provide in-depth insights into the organisational structures of exercise therapy care. Additionally, healthcare providers (HCPs) were interviewed to provide a process-oriented perspective on multidisciplinary care (i.e., medical care and supportive services). Heterogeneous participant selection was implemented to capture a board range of perspectives in cancer care.

### Participant selection and recruitment

The seven CCCs are key collaborators in the MOVE-ONKO project and share the common goal of implementing a care pathway for exercise therapy care for oncology patients in Germany (for further details on CCC characteristics, see Supplement II). Each CCC has a KI responsible for the exercise therapy care unit at their location. HCP participants were contacted and recruited by the seven KIs using postcards to distribute, containing QR codes, which allowed them to express interest in participating in an interview. To ensure a purposeful sampling approach [25], we provided KIs with information and postcards to select participants based on their experience of at least six months, their professional background within the context of exercise therapy care, and their level of care involvement. Purposeful sampling was chosen to capture a diverse range of perspectives and experiences. All interested HCPs where interviewed. Due to the recruitment process via the KIs, we do not have any information about refusal to participate. We interviewed seven KIs and 24 HCPs. Detailed participant characteristics are presented in Table 1.

**Table 1:** Participant characteristics

	KI (*n* = 7)	HCP (*n* = 24)
Age in years, *n* (%)		
< 29y	0 (0%)	1 (4.2%)
30–39y	2 (28.6%)	10 (41.7%)
40–49y	2 (28.6%)	6 (25%)
50–59y	1 (14.3%)	5 (20.8%)
60y >	2 (28.6%)	2 (8.3%)
Gender, *n* (%)^1^		
Female	3 (42.9%)	21 (87.5%)
Male	4 (57.1%)	3 (12.5%)
Experience in years, mean (SD)		15.3 (± 10.6)
Leadership in years, mean (SD)	7 (± 4.8)	
Professional groups (*n*)	Leaders of exercise therapy care units	Sports science (8); Physicians (5); Nurses (3); Psycho-oncology (2); Nutritional therapy (1); Social services (1); Other (4)

Key Informants (*KI*), Healthcare Providers (*HCP*), Standard deviation (*SD*)

^1^Non-participants identified as diverse

Fourteen of the 31 interviews were conducted face-to-face at CCCs to gain insights into their structures and facilities, while the remaining interviews took place online or by phone when site visits were not possible.

### Data collection

First, the KIs received a self-developed and pretested (using think-aloud interviews) postal questionnaire regarding organisational structures and patient care metrics (see Supplement II; further details on the survey are presented in the study protocol [21]). All data were self-reported by KIs or gathered with the support of the CCC’s controlling team. Second, all the KIs were invited to participate in a semi-structured interview and all recruited HCPs by the KIs were invited to an interview. All interviewees were fully informed about the aim of the study, the researchers’ interests, and their right to withdraw at any time. They were also assured that all data would be treated confidentially and anonymised. Informed consent was obtained from all participants. Each interview began with an overview of the study’s objectives and structure, and all interviews were recorded. Interviews were individually conducted mainly by SB (master’s degree in health economics, research assistant, trained in qualitative research, female) and partly by AT (master’s student, trainee in qualitative health services research, female). Field notes were taken by the researchers during the interviews.

Two interview guides (see Supplement III) were used to explore network characteristics, including network structures and dynamics. The KI interviews focused on structural preconditions, patient flow, staffing, and workload from a top-down perspective, whereas the HCP interviews examined collaboration in daily care from a bottom-up view. Both guides address network structures, with a focus on exercise therapy care and patient access.

The average interview duration was 33 min (min: 11:39; max: 1:08:45). The interviews were conducted in German, and no other individuals were present. They took place once between December 2023 and February 2024. Transcripts were not returned to participants. Data collection continued until thematic saturation was reached. The need for additional interviews was communicated during monthly meetings between the site study teams.

#### Data analysis

The audio recordings were transcribed and anonymised by SB, AT, and JK (master’s student in health services research, female). Names, locations, and site-specific details were replaced with numerical codes to ensure that interviewees could not be identified. The interview data were analysed via qualitative content analysis [26] with MAXQDA Analytics Pro 2022 (Version 22.8.0).

Over the course of four months, we held monthly meetings to refine the coding framework by writing memos and analysing themes related to patterns of care, care setting, and context. The codebook (see Supplement IV) was developed in three waves of coding. In each wave, each of the three researchers involved analysed four interviews, which were then discussed during monthly meetings. After the third wave, we double-coded 24 interviews and achieved an inter-coder reliability of 70%. The remaining interviews were subsequently analysed individually. Disagreement was discussed in the monthly meetings.

The main categories were initially derived from the interview guides based on core questions about the following subjects: Networks (intra-organisational, inter-organisational, and regional), communication, resources, facilities and existing care processes/pathways (see Supplement II). Subcategories were added inductively during the coding process by two researchers based on recurring patterns in the data (SB and AT/JK). The inductive approach was further used to specify unknown network patterns as it involved developing new subcodes from the data. This methodology extension followed three additional steps, with a focus on networks, whereby coded interviews represented actors as nodes and their relationships as edges [27]. Relationships were defined by the presence of two co-occurring codes on nodes and their described interaction. (1) Firstly, we identified actors involved in exercise therapy care, (2) Second, we coded their interactions as relational components, and (3) third, we analysed overarching patterns to define common principles of operation as they represent a shared logic of interactional behaviour within these provider networks [11].

The KIs’ questionnaires were analysed using SPSS. Since the qualitative data provided contextual data on participating CCCs only, integration of qualitative and quantitative data was not planned beyond acknowledging each CCC’s context.

## Results

This section begins by outlining the contextual care settings (including the CCCs’ patient flow) based on survey results as well as existing care structures and the development of exercise therapy care at the seven CCCs based on interview data. These contextual descriptions are then elaborated from a network perspective, focusing on network actors and their relationships, and are presented as common relational network patterns of care. These patterns are derived from the interview data (see Supplement IV).

### Contextual care settings at CCCs

#### Current care pathway

**Fig. 2:**
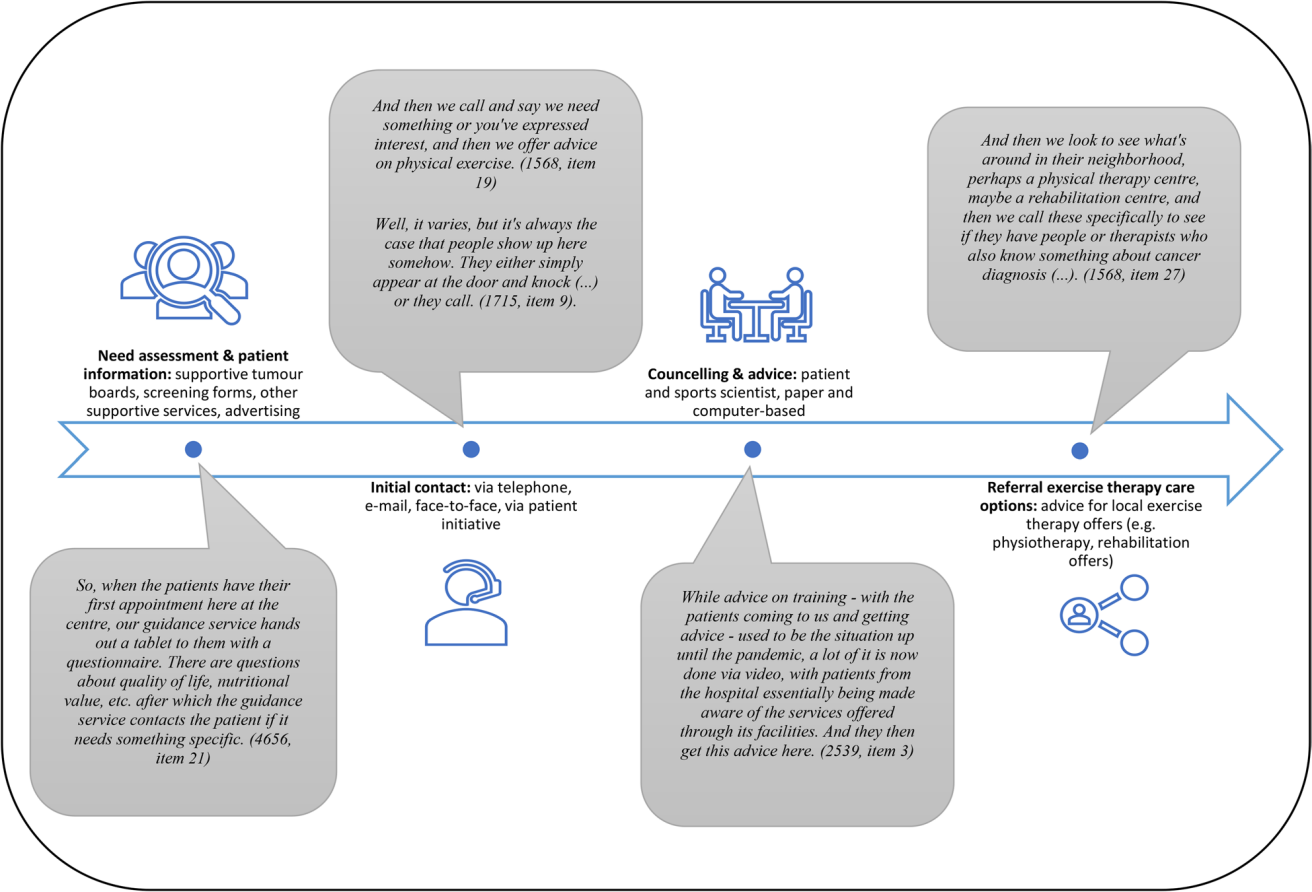
Current care process

All seven invited KIs completed the survey. The average patient flow (mean and standard deviation^1^) across the seven CCCs in 2023 was 6,836 (± 2971) cancer patients in medical care. Of these, 168 (± 137) received exercise counselling, and 111 (± 76) were referred to oncology exercise interventions (see Supplement II for detailed information). Thus, all CCCs already provide patients access to exercise therapy care. These care structures are described in the following section. The main category *Current Care Process* was further subdivided inductively by four subcodes (*Needs Assessment & Patient Information*,* Initial Contact*,* Counselling & Advice*,* Referral to Exercise Therapy Care Options*; see Fig. 2 and Supplement IV).

All interviewees described their current care pathways of patient access into exercise therapy, beginning with some form of needs assessment for exercise therapy care when patients start cancer treatment (see Fig. 2). Interviewees stated that patients complete a comprehensive screening form covering all supportive services or specific questionnaires to assess their needs. Alternatively, they may come across information during their clinic visits, such as flyers or brochures, that piques their interest. Patients then meet the exercise therapy care team (primarily sports scientists), either by reaching out themselves or through a referral from the medical care team. Following this initial contact, they are invited to a counselling session where they can ask questions, discuss risks and benefits, and identify suitable exercises. If they choose to participate, they are referred to appropriate exercise therapy offers.

#### Development of exercise therapy care

The interviews described the historical development of exercise therapy in cancer care as well as current care processes. The interviewees explained that, in the 1990 s, exercise therapy for cancer patients was still in its infancy in Germany. At that time, they recalled, many HCPs viewed cancer treatments as largely ineffective and therefore saw no need for exercise therapy. The interviewees themselves, however, described these views as reflective of the medical paradigm rather than their own opinions. Interviewees noted that the evidence base has expanded in the last 40 years and is now well established, yet exercise therapy care is still not consistently translated into practice. As a result, patient access to exercise therapy care, as well as the increase of availability and accessibility of exercise therapy care options, largely depends on the efforts of individuals, such as sports scientists and physicians. Interviewees pointed out that the exercise therapy care offered in CCCs is heavily reliant on individual contributions, highlighting the urgent need for better support and integration, as the following quote underlines:

I think what there is so far depends to an extreme extent on the commitment of individual people, and I think that’s a real shame. […]-And, as I said, without all that private commitment, none of the locations would be anywhere near where they are now. And, of course, I also hope this project will make things a bit easier, because it’s been a very rocky road here so far, despite a huge amount of backing and support. (4230, item 148)

Interviewees reported not only on how the current care process operates in Germany but also on the origins of exercise therapy. They remarked that some of these initiatives stemmed from sports medicine, where physicians specialised in sports medicine, acknowledged the important role of physical activity and exercise for cancer patients. As a result, physicians integrated specialised oncology exercise therapy care into their medical care and began collaborating with sports scientists. At the same time, the interviewees noted that exercise therapy care for cancer patients has also been developed within sports science. Professionals in this field typically enter through traditional academic pathways, including studying and completing a doctoral thesis. Over time, these careers have evolved towards a stronger focus on exercise therapy care for cancer patients, resulting in the first units of exercise therapy care in CCCs.

You would imagine a huge hall with 30 or 40 items of equipment. Well, it’s not like that at all. But it is a start. And, as I said, this wasn’t even possible a few years ago, but it’s been set up very quickly, so we can now offer around three training sessions per week. (1568, item 15)

### Principles of operation in exercise therapy care networks

**Fig. 3:**
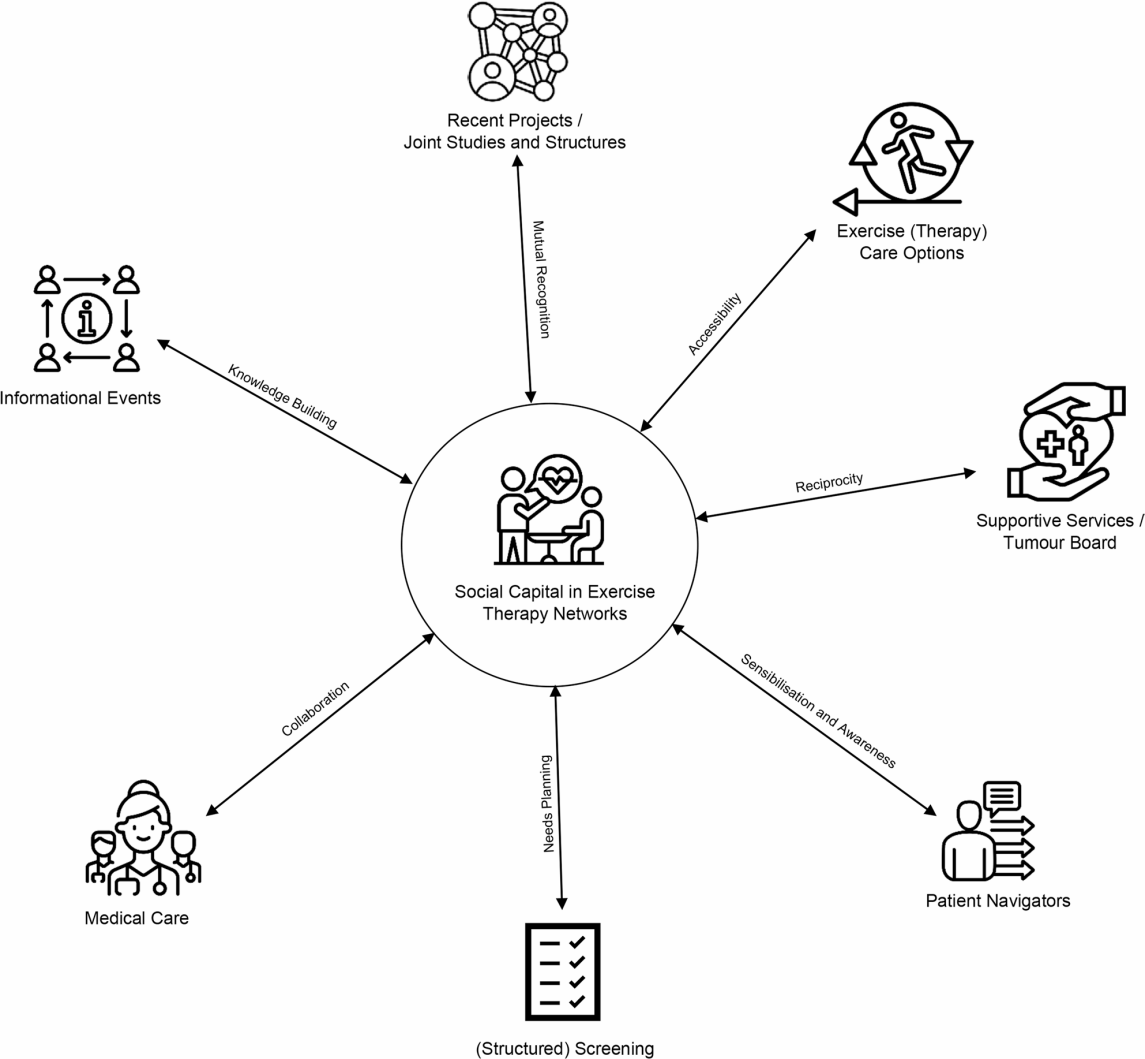
Principles of operation

The second and third steps of the analysis focused on understanding the actors and relationships within the current care networks for exercise therapy in cancer care (see Fig. 3). Based on the interview data and the inductively coded actors and relationships (see Supplement IV), overarching network patterns were derived from the data. From this, we identified seven principles of operation for the CCCs’ exercise therapy care networks and their social capital for further growth.

At the centre of Fig. 3, exercise therapy care is surrounded by the common relational structures of the seven CCCs (for further information see Supplement V). The seven principles are explained in the following sections.

#### Principle of reciprocity

The interviewees identified the shared relational structure as the cornerstone of these principles, illustrating it with the supportive tumour board in some CCCs and highlighting the board’s reciprocal nature (see Fig. 3). The interviewees described the supportive tumour board as a forum where all supportive services meet regularly to discuss patients and coordinate care, mirroring the structure of a clinical tumour board but with a focus on supportive services. Exercise therapy care providers tend to work closely with other supportive care teams, commonly coordinating their offerings and supporting one another.

Yes, and apart from that, information is exchanged extensively with other departments via the supportive tumour board, including all the support areas such as of psycho-oncology, physical exercise and nutrition. (8303, item 19)

#### Principle of sensibilisation and awareness

Existing patient navigators were described in some CCCs as being primarily responsible for guiding patients to appropriate counselling services and partly bridging the gap between medical care and supportive services. In some cases, patient navigators also take on the task of managing screening forms for all supportive services. Thus, relational interaction between all services plays a key role in strengthening coordinated care, integrating exercise therapy care into the general cancer care structure, and ensuring patient access by raising awareness of the topic through screening forms.

So, the guidance team’s [patient navigators] main job is to refer patients. That means to our support services, i.e. all the areas that have nothing to do with medical care. Nutritional counselling for example. Psycho-oncological counselling, specifically in the area of exercise therapy counselling […] That’s completely new now. And social counselling. (4563, item 5)

#### Principle of needs planning

The interviewees explained that screening patients at the start of supportive care services helps ensure that exercise therapy counselling meets individual patient needs before. Interviewees highlighted the timing and repetition of such screenings as important. This screening is sometimes carried out by nurses acting as patient navigators, by sports scientists themselves, or by medical staff (e.g., nurses and doctors) during medical treatments to facilitate patients access to exercise therapy care. In the absence of a structured screening process, exercise therapy relies on the interest and initiative of professionals in other disciplines to refer patients to exercise therapy care.

These are mainly in our area, for example, there are some who specialise in patients after therapy [special form of therapy] and they also do a bit of everything and are already involved in the guidance side. So, they sort out whether the patient has a psychological problem, a nutritional problem, or an issue with medication. That means they do a bit of screening, and it also seemed most sensible to us to train them as physical exercise guides, since they have long-term contact with the patients and also do a bit of screening for the other counselling services. (6533, item 37)

#### Principle of collaboration

The interviewees explained that exercise therapy care for oncology patients originated in sports sciences and sports medicine, but its development has relied primarily on the personal commitment of practitioners and specialists to improving patient care. The interviewees observed that a collaborative support structure has since emerged between the two disciplines, recognizing each side’s contributions and combining their resources to provide more comprehensive support for patients.

For example, some exercise therapy counselling is provided through joint appointments with patients that involve both sports scientists and physicians. This means that screening and counselling, as well as the gathering of more medical information, can be performed in one appointment. Interviewees also stated that counselling at other locations involves individual consultations by sports scientists. If necessary, any concerns about side-effects are clarified during case discussions or referred to a physician’s consultation for further evaluation. Alternatively, it may become the patient’s responsibility to seek additional information during their next medical appointment to rule out any contraindications for exercise therapy care. Nevertheless, interviewees underscored that a strong connection between sports science and sports medicine is highly beneficial.

In fact, all this is done entirely via the outpatient clinic. So, for the patients, it’s a normal health insurance benefit for those with statutory insurance. As we also carry out a routine examination - resting ECG, blood pressure measurement, height, weight, and always involving a doctor - this is actually billed simply and quite normally through the outpatient clinic, via the health insurance companies. And the fact that there is always a sports scientist in attendance is not currently financed in any way, as far as I’m aware. (4711, item 7)

#### Principle of knowledge-building

In addition to actively screening patients, knowledge development among medical care providers was described as a functional principle associated with positive synergistic effects. For example, internal training sessions and informational events helped physicians and nurses recognise exercise therapy care as a form of supportive care, leading them to recommend it during ward rounds and daily patient care. While medical care providers are highly interested in exercise therapy care, they rely on access to information and knowledge about exercise therapy care options.

So, there’s a lot of interest, as well as a great deal of uncertainty, and I feel there is also a very high level of uncertainty on the part of the doctors, with this also being passed on to the patients. (1715, item 17)

Even though informational events exist, interviewees noted that personnel resources are limited, and nurses and physicians often lack the time to convey all necessary information during ward rounds. Additionally, informational events rely on exercise therapy specialists (e.g., sports scientists) and are time-consuming and require frequent repetition throughout the year due to staff turnover and physician rotation during residency.

#### Principle of mutual recognition

Interviewees asserted that the shared history within a CCC facilitates access to interdisciplinary consultations involving not only sports medicine but also oncologists, radiologists, and internists. As a result, the benefits of shared structures and past projects within a care network have improved access to medical advice (e.g., in managing bone metastases) and ensured medical care support by referring patients to exercise therapy care. Despite strong evidence, the interviewees stated that exercise therapy care remains dependent on medical care support and needs to be integrated into existing structures. Medical care therefore continues to strongly influence patient expectations, as the following quotation illustrates:

Yes, so here at the hospital, as a result of these consultations, or since this started in 2019, links with the treating departments, such as radiation oncology, gynaecology, and neuro-oncology, have increased. And this makes a lot of patients aware of what is offered here. Before, it was actually the case that I repeatedly had people here who said that if they hadn’t been contacted by him or her or if they hadn’t discovered it by chance, they wouldn’t even have known about it. (9428, item 15)

#### Principle of accessibility

During counselling sessions, interviewees emphasised the importance of having a portfolio of available options to offer exercise therapy care that are both geographically accessible and aligned with the patient’s interests. Ideally, these programmes should be led by professionals with extensive experience and specialised training in oncology. Furthermore, when oncology patients share their experiences, it positively reinforces the CCC’s reputation and overall impact.

So, after this initial impetus, we quickly observed this snowball effect of patients starting to communicate with each other and then also launching other initiatives among themselves and also going swimming or the like. Yes, we are also aware that the group experience is particularly important, because every patient needs to adapt accordingly. So, I’m not necessarily the one who’s worst off. And the guy, he can do it too, so I also have to be able to. So, it’s an important group dynamic that plays a role […]. (3512, item 3)

## Discussion

At the seven CCCs, exercise therapy care provider networks operate according to seven relational principles that guide the development and integration of exercise therapy care in these centres. The major results of the interviews can be summarised as following. First, social capital is built on prior relationships, either at individual levels through shared academic experiences or at the institutional level through recent collaborative projects. Exercise therapy care networks draw on existing social ties to stabilise and sustain their services. Second, exercise therapy care networks are driven by individual commitment and a shared belief in the long-term integration of exercise therapy care into routine care. As a result, the networks remain unstable and insecure as long as no regulated care structures formalise their work. Third, exercise therapy services benefit reciprocally from their integration into the broader network of supportive care services within oncology care. In addition to medical oncology care, patients gain access to nutritional counselling, psycho-oncology, social services, and exercise therapy through interconnected service structures. These network ties play crucial roles in advancing and embedding exercise therapy within routine care. With the exception of psycho-oncology services [28, 29], most supportive care services are not yet formally integrated into the oncology care system in Germany. This lack of formal anchoring suggests a strong rationale for closer collaboration among disciplines. Current network relational patterns range from informal hallway exchanges to more structured formats, such as supportive tumour boards; this finding is supported by Voland et al. (2023), who evaluated network-building in regional exercise therapy networks. They determined that collaborative networks with common goals enable the involvement of professional actors from different operational fields as facilitators of patients’ access to exercise therapy care [30], which is in line with the international research perspective. The American College of Sports Medicine (ACSM) guidelines [4] highlight that establishing professional relationships across provider networks enables cancer patients to be referred efficiently between care services (e.g., from medical care to exercise therapy care). This underscores the importance of HCPs being aware of available exercise therapy options and patients’ individual needs to optimise treatment planning.

In addition to practical guidelines, research has played a crucial role in the development of exercise therapy. Galvão et al. (2025) traced the history of exercise therapy care research and noted that exercise oncology research gained visibility through stronger study designs, systematic reviews, and publications in leading oncology journals, which in turn informed global clinical recommendations [3]. At the same time, conceptual frameworks for physical activity provided structured approaches to organizing this emerging field [3], laying the foundation for evidence-based practice and guiding the implementation of exercise therapy in clinical settings.

Social capital within these networks facilitates the overarching goal of exercise therapy provision, namely delivering counselling and services to a broad patient population and thereby improving access. In this context, networks emerge as crucial resources for realisation of sustainable care pathways in exercise therapy care. This aligns with the general literature on organisational collaboration, in which goals and visions reinforce sustainable change and need to be addressed in healthcare networks [31]. Similarly, in organisational collaboration, shared goals and visions have been shown to support long-term change and should be actively fostered in healthcare networks [31].

A similar implementation initiative to MOVE-ONKO was tested in Australia for its acceptance and feasibility [32]. In this case, researchers interviewed patients, oncologists, and exercise professionals and found that the implemented referral pathway into exercise therapy relies on ‘numerous behaviour change principles’ (p. 6) and reducing barriers to accessing or increasing knowledge about exercise therapy [32].

In the United Kingdom, exercise therapy has recently become more professionalised through the introduction of clinical exercise physiology as a distinct profession within the healthcare system [33]. Over four years, the authors [33] observed that this new role became increasingly formalised [33]. In this context, care networks within healthcare organisations are crucial resources for the implementation of sustainable care pathways in exercise therapy care. Additionally, a formal regulation of exercise therapy in general would be a valuable resource for secured care structures in Germany.

Looking ahead to the implementation of the MOVE-ONKO project, with its structured care pathways and the development of regional networks, we emphasise that current operational principles provide a foundation for further development of exercise therapy care. Especially in a previously unregulated area such as exercise therapy, bottom-up solutions and strategies, which are usually based on individual operationalised relationships, are first steps for larger solutions. Thus, social relationships need to be further considered as a research object in exercise therapy care networks [34].

### Limitations

This study presents several limitations to consider. First, the inclusion of the seven CCCs and their participants was determined by the nature of the project. Only highly specialised CCCs and established exercise therapy care units were included in the MOVE-ONKO project and its evaluation. Thus, the results may not be transferable to smaller and less specialised centres. Second, the qualitative interviews with HCPs were constrained by the sampling strategy in which the KIs selected the HCPs for us. Although we provided the KIs with information on sampling strategies, they may have prioritised high-performing respondents. Nevertheless, a broad range of participants was included, allowing us to gain deep insights into each CCC. Third, some authors of this paper were also included in the interview sample. The contribution of the interviewed individuals who were also authors was limited to reviewing and editing this manuscript as well as to the overall conceptualisation of the study. They were not involved in data analysis. Fourth, not all CCCs were visited in person during the interviews. Some interviews were conducted online or via telephone. As a result, the CCCs appeared highly heterogeneous in terms of facilities, labels, and naming conventions. This made it time-consuming to analyse their current structures of care and common operational principles.

An exploratory qualitative network analysis emphasising relationships as a relational approach proved to be a suitable method for examining such an unstructured field of care and may be useful in other research fields. However, networks always represent a snapshot of a specific situation and do not fully capture all care dynamics and relational structures. Therefore, the potential for further network research based on quantitative and qualitative data is worth exploring.

## Conclusion

Informal but foundational networks in exercise therapy care in Germany have already been established and must now be stabilised and sustained to support further development of holistic patient access to exercise therapy care. Our results suggest that social capital, supported by the seven principles of operation, can be an important lever for driving changes in exercise therapy care. Simultaneously, in healthcare, the successful implementation of innovation heavily depends on its provider actors, which must be considered when planning implementation strategies. While existing structures are already in place, the key moving forward is to effectively build on these foundations and implement tailored strategies that lead to sustainable, long-term accessibility changes within the organisation’s context. The seven principles may be adapted and applied to other care contexts and settings.

As long as the implementation gap in exercise therapy care persists, social networks in exercise therapy care and established patient pathways will remain informal and are therefore potentially vulnerable. Formal regulations and financial security can strengthen those networks to ensure stability and sustainability in enabling patient access to exercise therapy care.

The results shown here represent the pre-implementation phase of the MOVE-ONKO project. The next steps, as outlined in the study protocol [21], involve implementing structured care pathways in the seven CCCs by the HCPs, beginning with a brief screening and awareness-raising session by trained exercise oncology guides (e.g. nurses). This will be followed by a counselling session with an exercise oncology specialist (e.g. sports scientists), who will provide referrals to exercise therapy options. The intervention draws on network-building as a key determinant of implementation success.

## Supplementary Information

Supplementary Material 1.

## Data Availability

Additional data will be available on request.

## Abbreviations

ACSM: American College of Sports Medicine
CCC: Comprehensive Cancer Centres
CICI: Context and Implementation of Complex Interventions Framework
HCP: Healthcare Providers
KI: Key Informants
MOVE-ONKO: Multi–professional care pathway and network for the promotion of needs–oriented, resident–oriented exercise therapy for oncology patients (Multiprofessionelle Versorgungsstruktur und Netzwerk zur Förderung bedarfsorientierter, wohnortnaher Bewegungstherapie von onkologischen Patient*innen)

## Footnotes

1 Numbers were rounded up if the decimal was 0.5 or higher.

## References

[1] ZfKD R. Koch-Institut. Anzahl der Krebsneuerkrankungen und durch Krebs verursachte Todesfälle in Deutschland nach Geschlecht im Jahr 2022: [Graph]. 2024. https://de.statista.com/statistik/daten/studie/30563/umfrage/jaehrliche-krebserkrankungen-und-todesfaelle-in-deutschland/. Accessed 19 Dec 2024.

[2] Schmidt T, Süß P, Schulte DM, Letsch A, Jensen W. Supportive care in oncology-from physical activity to nutrition. Nutrients. 2022. 10.3390/nu14061149. 35334806 10.3390/nu14061149 PMC8954702

[3] Galvão DA, Courneya KS, Lucia A, May AM, Mustian K, Warner AB, et al. History informing the future of exercise oncology. J Natl Cancer Inst Monogr. 2025;2025:306–14. 10.1093/jncimonographs/lgaf025. 40828163 10.1093/jncimonographs/lgaf025 PMC12363226

[4] Schmitz KH, Campbell AM, Stuiver MM, Pinto BM, Schwartz AL, Morris GS, et al. Exercise is medicine in oncology: engaging clinicians to help patients move through cancer. CA Cancer J Clin. 2019;69:468–84. 10.3322/caac.21579. 31617590 10.3322/caac.21579 PMC7896280

[5] Courneya KS, Friedenreich CM. Physical activity and cancer control. Semin Oncol Nurs. 2007;23:242–52. 10.1016/j.soncn.2007.08.002. 18022052 10.1016/j.soncn.2007.08.002

[6] Voland A, Lohmann A, Ansmann L, Wiskemann J. Barriers and facilitators for the implementation of exercise oncology provision in Germany: a multilevel, mixed-methods evaluation of the network OnkoAktiv. Eur J Cancer Care. 2023;2023:6270049. 10.1155/2023/6270049.

[7] Wiskemann J, Köppel M, Voland A, Rosenberger F, Biazeck B, Wegener A, et al. Implementation von Sport- und Bewegungstherapie in die onkologische Routineversorgung. TumorDiagnostik & Therapie. 2020;41:306–10.

[8] Alderman G, Semple S, Cesnik R, Toohey K. Health care professionals’ knowledge and attitudes toward physical activity in cancer patients: a systematic review. Semin Oncol Nurs. 2020;36:151070. 10.1016/j.soncn.2020.151070. 33010981 10.1016/j.soncn.2020.151070

[9] Höh J-C, Schmidt T, Hübner J. Physical activity among cancer survivors-what is their perception and experience? Support Care Cancer. 2018;26:1471–8. 10.1007/s00520-017-3977-0. 29168034 10.1007/s00520-017-3977-0

[10] Granovetter MS. The strength of weak ties. Am J Sociol. 1973;78:1360–80.

[11] Hollstein B. Personal network dynamics across the life course: A relationship-related structural approach. Adv Life Course Res. 2023;58:100567. 38054867 10.1016/j.alcr.2023.100567

[12] Parnell JM, Robinson JC. Social network analysis: presenting an underused method for nursing research. J Adv Nurs. 2018;74:1310–8. 10.1111/jan.13541. 29444337 10.1111/jan.13541 PMC6033272

[13] Provan KG, Kenis P. Modes of network governance: structure, management, and effectiveness. J Public Adm Res Theor. 2007;18:229–52. 10.1093/jopart/mum015.

[14] Glegg SMN, Jenkins E, Kothari A. How the study of networks informs knowledge translation and implementation: a scoping review. Implement Sci. 2019;14:34. 10.1186/s13012-019-0879-1. 30917844 10.1186/s13012-019-0879-1 PMC6437864

[15] Schot E, Tummers L, Noordegraaf M. Working on working together. A systematic review on how healthcare professionals contribute to interprofessional collaboration. J Interprof Care. 2020;34:332–42. 10.1080/13561820.2019.1636007. 31329469 10.1080/13561820.2019.1636007

[16] Gernert M, Fohr G, Schaller A. Network development in workplace health promotion – empirically based insights from a cross-company network promoting physical activity in Germany. BMC Public Health. 2024;24:1560. 10.1186/s12889-024-19025-4. 38858699 10.1186/s12889-024-19025-4 PMC11165875

[17] Pierre Bourdieu. Forms of capital. Economic Sociol. 2002;3:60–74.

[18] Putnam RD. Bowling alone: america’s declining social capital. In: Crothers L, Lockhart C, editors. Culture and politics: A reader. New York: Palgrave Macmillan US; 2000. pp. 223–34. 10.1007/978-1-349-62965-7_12.

[19] Lin N. Chapter 4 buidling a network theory of social capital: social Capital, social support and stratification. In: Cheltenham, UK: Edward Elgar Publishing; 2019. 10.4337/9781789907285.00009.

[20] Pfadenhauer LM, Gerhardus A, Mozygemba K, Lysdahl KB, Booth A, Hofmann B, et al. Making sense of complexity in context and implementation: the Context and Implementation of Complex Interventions (CICI) framework. Implement Sci. 2017;12:21. 10.1186/s13012-017-0552-5. 28202031 10.1186/s13012-017-0552-5 PMC5312531

[21] Blütgen S, Krug K, Graf K, Betz U, Böhm J, Jäger E, et al. Implementation and evaluation of a multi-professional care pathway and network for the promotion of needs-oriented, resident-oriented exercise therapy for oncological patients (MOVE-ONKO): protocol of a mixed-methods cohort study. BMC Cancer. 2025;25:458. 10.1186/s12885-025-13797-7. 40082847 10.1186/s12885-025-13797-7 PMC11908027

[22] Grosso D, Aljurf M, Gergis U. Building a Comprehensive Cancer Center: Overall Structure. In: Aljurf M, Majhail NS, Koh MBC, Kharfan-Dabaja MA, Chao NJ, editors. The Comprehensive Cancer Center: Development, Integration, and Implementation. Cham (CH); 2022. pp. 3–13. 10.1007/978-3-030-82052-7_2.

[23] Tong A, Sainsbury P, Craig J. Consolidated criteria for reporting qualitative research (COREQ): a 32-item checklist for interviews and focus groups. Int J Qual Health Care. 2007;19:349–57. 10.1093/intqhc/mzm042. 17872937 10.1093/intqhc/mzm042

[24] Mielke J, Leppla L, Valenta S, Zullig LL, Zúñiga F, Staudacher S, et al. Unraveling implementation context: the Basel approach for contextual analysis (BANANA) in implementation science and its application in the smile project. Implement Sci Commun. 2022;3:102. 10.1186/s43058-022-00354-7. 36183141 10.1186/s43058-022-00354-7 PMC9526967

[25] Palinkas LA, Horwitz SM, Green CA, Wisdom JP, Duan N, Hoagwood K. Purposeful sampling for qualitative data collection and analysis in mixed method implementation research. Adm Policy Ment Health. 2015;42:533–44. 10.1007/s10488-013-0528-y. 24193818 10.1007/s10488-013-0528-y PMC4012002

[26] Rädiker S, Kuckartz U. Analyse qualitativer Daten mit MAXQDA: Springer; 2019.

[27] Pokorny JJ, Norman A, Zanesco AP, Bauer-Wu S, Sahdra BK, Saron CD. Network analysis for the visualization and analysis of qualitative data. Psychol Methods. 2018;23:169–83. 10.1037/met0000129. 28569530 10.1037/met0000129

[28] Kusch M, Labouvie H, Schiewer V, Talalaev N, Cwik JC, Bussmann S, et al. Integrated, cross-sectoral psycho-oncology (isPO): a new form of care for newly diagnosed cancer patients in Germany. BMC Health Serv Res. 2022;22:543. 10.1186/s12913-022-07782-0. 35459202 10.1186/s12913-022-07782-0 PMC9034572

[29] Hui D, Hoge G, Bruera E. Models of supportive care in oncology. Curr Opin Oncol. 2021;33:259–66. 10.1097/CCO.0000000000000733. 33720070 10.1097/CCO.0000000000000733 PMC8641044

[30] Voland A, Köppel M, Peters S, Wiskemann J, Wäsche H. Exploring the organisational structure of networks for exercise oncology provision: a social network analysis of onkoaktiv. BMC Health Serv Res. 2023;23:555. 10.1186/s12913-023-09572-8. 37244985 10.1186/s12913-023-09572-8 PMC10225106

[31] Seaton CL, Holm N, Bottorff JL, Jones-Bricker M, Errey S, Caperchione CM, et al. Factors that impact the success of interorganizational health promotion collaborations: a scoping review. Am J Health Promot. 2018;32:1095–109. 28587471 10.1177/0890117117710875

[32] Caperchione CM, English M, Sharp P, Agar MR, Phillips JL, Liauw W, et al. Exploring the practicality and acceptability of a brief exercise communication and clinician referral pathway in cancer care: a feasibility study. BMC Health Serv Res. 2023;23:1023. 10.1186/s12913-023-10003-x. 37740170 10.1186/s12913-023-10003-x PMC10517509

[33] Jones H, Crozier A, George K, Miller G, Whyte GP, Rycroft J, et al. Establishment of clinical exercise physiology as a regulated healthcare profession in the UK: a progress report. BMJ Open Sport Exerc Med. 2024;10:e002033. 10.1136/bmjsem-2024-002033. 38911478 10.1136/bmjsem-2024-002033 PMC11191777

[34] Vacchiano M, Hollstein B, Settersten RA, Spini D. Networked lives: probing the influence of social networks on the life course. Adv Life Course Res. 2024;59:100590. 10.1016/j.alcr.2024.100590. 38301296 10.1016/j.alcr.2024.100590

