# Supplementary material for: ‘It only works if we coordinate our work, that’s what we need’: a relational qualitative network analysis of exercise therapy in oncology care in Germany
Source: BMC Cancer. 2025 Nov 29;25:1845. doi: 10.1186/s12885-025-15326-y (PMC12670853; doi:10.1186/s12885-025-15326-y)
Supplement: Supplementary file 1 — Supplementary Material 1. [file 12885_2025_15326_MOESM1_ESM.docx]

**Supplement I: COREQ-Checklist**

**
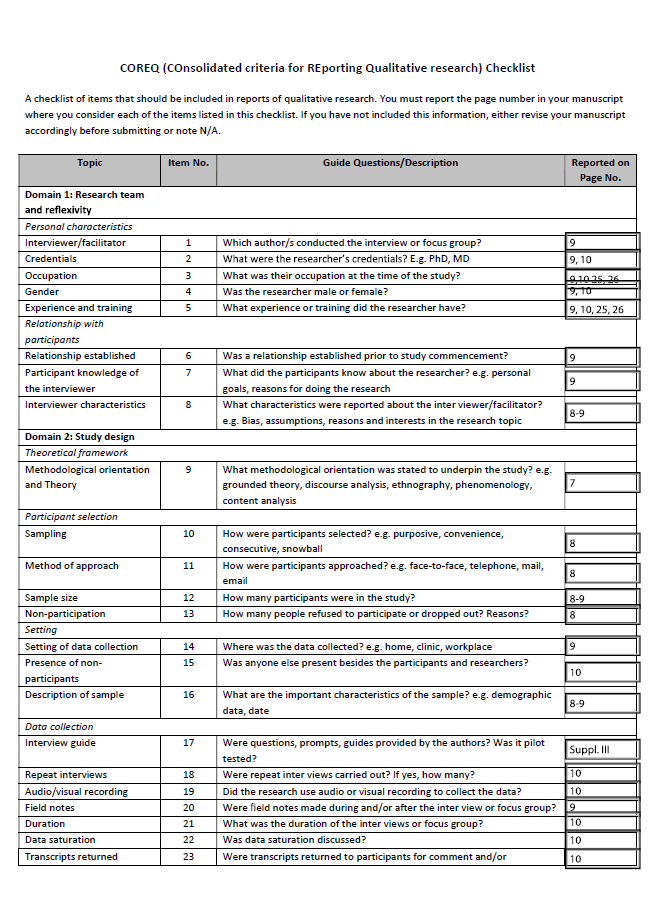
**

**
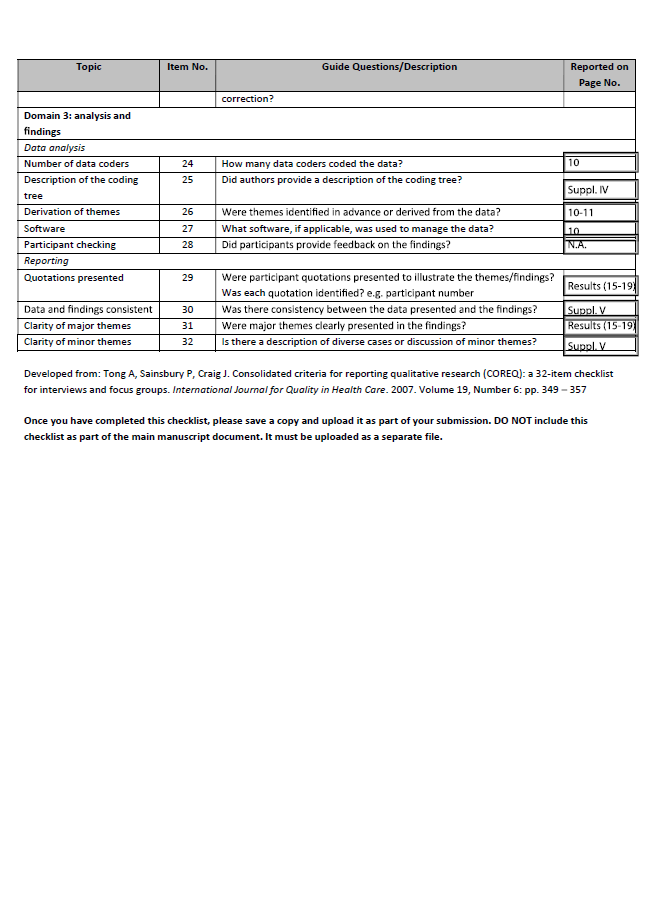
**

**Supplement II: Description of CCCs**

| Location | Region | Hospital Bed Capacity (2023) | Case Mix Index (2023) | Total Staff (2023) | Inpatients (and day treatments^[[1]](#footnote-1)^) (2023) | Outpatients (2023) | Teaching status/  Ownership | Positioning of oncology exercise therapy |
| --- | --- | --- | --- | --- | --- | --- | --- | --- |
| A | Rhineland-Palatinate | 1 665 | 1.22 | 8 726 | 61 358 | 276 163 | Public financed university hospital | Exercise therapy is located at the UCT^[[2]](#footnote-2)^ outpatient clinic and is part of the Institute for Physical Therapy, Prevention, and Rehabilitation |
| B | Berlin | 3 293 | 1.29^[[3]](#footnote-3)^ | 23 479 | 137 825 | 787 757 | Public financed university hospital | University hospital: Oncology Exercise therapy is part of the Department of Sports Medicine (which resulted from the merger of the Sports Medicine departments of Humboldt University of Berlin and Charité) |
| C | Baden-Württemberg | 2 179 | 1.26 | 15 127 | 89 438 | 970 646 | Public financed university hospital | University hospital: Oncology Exercise therapy (here: sports oncology) is located at the Department of Internal Medicine I (Haematology, Oncology, and Stem Cell Transplantation) and is part of the ITZ^[[4]](#footnote-4)^ |
| D | Hesse | 503 | NA | 2.400 | 18 100 | 33 400 |  | Clinic for specialised care and academic teaching hospital of the Goethe University. Part of the University Cancer Centre, UCT Frankfurt-Marburg: Oncology exercise therapy is located at the department of Oncology and Haematology (inpatient and outpatient). UCT Frankfurt-Marburg: Was founded in 2008 in collaboration with the University Hospital Frankfurt, the Faculty of Medicine of Goethe University, and Krankenhaus Nordwest. |
| E | Baden-Württemberg | 1 637 | NA | 10 956 | 73 675 | 406 901 | Public financed university hospital | Oncology exercise therapy is located in sports medicine. |
| F | Saxony | 1 410 | 1.265 |  | 53 259  (+7900^3^) | 237 503 | Public financed university hospital | Oncology exercise therapy is affiliated with the NCT and is part of the Prevention Centre (develops and disseminates programs for primary and tertiary cancer prevention) |
| G | Baden-Württemberg | 2 599 | 1.28 | 14 057 | 88 557 | 1 244 103 | Public financed university hospital | Oncology Exercise therapy is affiliated with the NCT. |
| A Quelle: [Universitätsmedizin Mainz \| Daten und Fakten](https://www.unimedizin-mainz.de/ueber-uns/daten-und-fakten.html) (Abgerufen am 15.01.2025)  B Quelle: [Publikationen: Charité – Universitätsmedizin Berlin](https://www.charite.de/die_charite/mediathek/publikationen/) (Abgerufen am 15.01.2025)  C Quelle: [Jahresbericht 2023 \| Universitätsklinikum Freiburg](https://www.uniklinik-freiburg.de/jahresbericht-2023.html) (Angerufen am 15.01.2025)  D Quelle: [Profil und Organisation](https://www.krankenhaus-nordwest.de/unternehmen/profil-und-organisation) (Abgerufen am 15.01.2025)  E Quelle: [Daten und Fakten \| Universitätsklinikum Tübingen](https://www.medizin.uni-tuebingen.de/de/das-klinikum/daten-und-fakten) (Abgerufen am 15.01.2025)  F Quelle: [Das Universitätsklinikum Carl Gustav Carus Dresden — Deutsch](https://www.uniklinikum-dresden.de/de/das-klinikum) (Abgerufen am 14.01.2025)  G Quelle: [UKHD Jahresbericht 2023 \| Universitätsklinikum Heidelberg](https://bericht.ukhd-mfhd.de/2023/) (Abgerufen am 14.01.2025) | | | | | | | | |

**Supplement III: Interview guidelines**

**Interview guidelines for qualitative interviews in the MOVE-ONKO project**

**
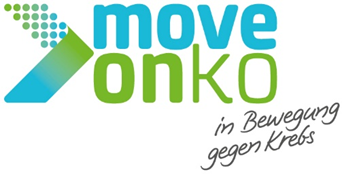
**


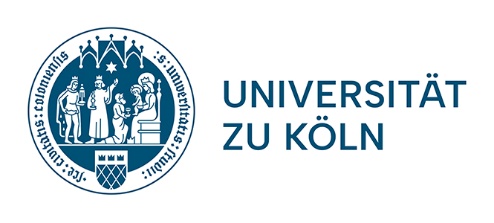

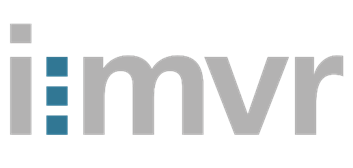
 **General Information**

| Aim | Survey of processes, structures and needs with regard to the implementation of exercise therapy in the participating facilities prior to implementation |
| --- | --- |
| Duration of interviews | 30-60 minutes |
| Venue | Comprehensive Cancer Centre (CCC) / telephone / video conference |
| Preparation | Record device |
| Arrival of participants | Hand out study information and consent forms again, answer questions if necessary |

**Part 1: Introduction, project presentation, preparation**

| Introduction | Welcome, thank you very much for taking the time for this interview as part of the MOVE-ONKO project.  My name is XY and I am XY. Together with my colleague XY, we are responsible for the evaluation of the MOVE-ONKO project at the Chair of Medical Sociology at the Institute of Medical Sociology, Health Services Research and Rehabilitation Sciences at the University of Cologne. |
| --- | --- |
| Project Introduction | Before we start the interview, I would like to briefly tell you about the aim of our project and this interview.  The aim of the MOVE-ONKO project is to implement a previously missing, relevant multi-professional care structure for the promotion and therapy of physical activity as an interface between oncological care and existing or developing exercise therapy services in the area.  Phase 1: Today's interview is about the current situation (the status quo), i.e. the processes and aspects relating to the implementation of exercise therapy in your institution before the start of the intervention.  The interview usually takes about 30 to 60 minutes, which can vary depending on the course of the conversation. If you need a break during the interview, please let us know. |
| Recording and data protection | Your answers will of course be treated confidentially and anonymously, i.e. it will not be possible for outsiders to recognise who you are. We would like to emphasise that we are independent scientists and are interested in your experiences. You can answer all questions openly and honestly.  Your participation in the interview is voluntary and you may stop at any time. You may also not answer questions. In general, there are no right or wrong answers to any of the questions I will ask. If you have any questions during the interview, please feel free to ask them.  The interview will be recorded using a digital recorder, later transcribed, anonymised and finally analysed.  In this context, you have in front of you two copies of the study information, including the consent form, which we sent you digitally seven days ago. This contains all the relevant information about the study and data processing in more detail.  Do you have any questions about the information?  Then I would like to ask you to sign the declaration of consent and submit a copy to us. |
| Start | Do you have any questions?  Then let's start the recording now. |

**Part 2:** **Interview guide (status quo) for healthcare providers**

| **Subject** | **Core questions** | **In-depth questions** | **Notes** | |  |
| --- | --- | --- | --- | --- | --- |
| **Personal background** | **How did you come to the CCC and the exercise therapy?** | - What is your current role in exercise therapy in oncology? - How are you qualified in the field of exercise therapy? (studies and further training) |  | |  |
| **Everyday life at work** | **Could you tell us a bit about your daily work routine?** | - What are your responsibilities and functions? - How do you build confidence in your therapeutic practice? - How do you stay informed about exercise and cancer care? - What is your approach to developing therapeutic recommendations in the context of exercise therapy? |  | |  |
| **Personal experiences** | **What experiences have you had so far with exercise therapy care, could you tell us about them?**  (Optional: to be omitted if already covered when discussing everyday practice) | - To what extent do you already incorporate exercise-based approaches into your therapeutic work? - In your opinion, what is still lacking in this regard? - What challenges have you encountered? |  | |  |
| **Existing Care Pathways (and Adherence)** | **How are patients currently made aware of and referred to exercise therapy at your CCC?** | - How do you inform patients about exercise therapy? - How do you motivate patients to participate? - How are the available exercise therapy services accessed and used by patients? - How do patients make use of the available exercise therapy services? - How is the exercise therapy provision organised within your institution? - Where do you see potential for improvement in the current care processes? - What specific services or programmes do you currently offer in the field of exercise therapy? |  | |  |
| **Facilities and Equipment** | **Could you describe where exercise therapy consultations usually take place and what technical tools or resources you use during these sessions?** | - Are there any specific areas or resources where you see a need for improvement? - How satisfied are you with the facilities and equipment available at your institution to deliver and coordinate exercise therapy for patients? | |  | |
| **Optional: Do you intend to become an exercise oncology guide?** | | | |  | |
| **Training / Qualification** | **What are your expectations regarding the new role and the training?** | - What sparked your interest in the training or in taking on this new role? - Do you have any additional or specialised qualifications that might support you in your role? - What do you think would help you to familiarise yourself with this role? | |  | |
| **Changes** | **What changes do you anticipate in your daily work routine as a result of taking on this new role?** | - Do you think the new role might bring added pressure or stress to your current workload? - To what extent do you expect the role to require additional time or effort? | |  | |
| **Networks and interdisciplinary communication, work atmosphere** | **Who do you have contact with in your day-to-day work, who do you work with?** | - With external training centres? - In the CCC? - Who is missing/who else would you like to work with? - If patients in your CCC receive a recommendation for exercise therapy, to what extent do the external trainers follow your recommendations? | |  | |
|  | **Can you tell me how you perceive the general working atmosphere and cooperation in your organisation?** | - How do colleagues interact with each other? - How is the interdisciplinary interaction? - Can you tell me something about working with colleagues XY? - What role do hierarchies play in your organisation? | |  | |
| **The study's expectations** | **What do you hope to achieve with the MOVE ONKO project?**  (Optional: Omit if expectations have already been told in the training section) | - What is your personal motivation for participating in the project? - What do you need before starting the implementation? - Where do you see difficulties? | |  | |
| **Wishes for the future** | **What do you wish for the future of your institution/therapy programme?** | - Future wishes, possibly related to exercise therapy or in general, communication and cooperation internally/externally, spatial equipment and IT, etc. | |  | |
| **Attitude towards exercise** | **What role does exercise play for you personally?** | - How do you integrate exercise into your everyday life? | |  | |
| Is there anything else you would like to pass on to us// is there anything else we haven't talked about yet that you would like to tell us? | | | | | |
| **End and goodbye** | | | | | |

**Part 2: Interview guide (status quo) for key informants**

| **Subject** | **Core questions** | **In-depth questions** | **Notes** | |  |
| --- | --- | --- | --- | --- | --- |
| **Personal background** | **How did you come to the CCC and the exercise therapy?** | - What is your current role in exercise therapy in oncology? - How are you qualified in the field of exercise therapy? (studies and further training) |  | |  |
| **Existing Care Pathways (and Adherence)** | **How are patients currently made aware of and referred to exercise therapy at your CCC?** | - How do you inform patients about exercise therapy? - How do you motivate patients to participate? - How are the available exercise therapy services accessed and used by patients? - How do patients make use of the available exercise therapy services? - How is the exercise therapy provision organised within your institution? - Where do you see potential for improvement in the current care processes? - What specific services or programmes do you currently offer in the field of exercise therapy? |  | |  |
| **Professional requirements and participation** | **Who in your CCC is responsible for addressing and referring people to exercise therapy and what qualifications do they have?** | - What are the qualifications of service providers in your CCC who take on tasks in the approach/referral to exercise therapy? - Which roles or competences in the team/CCC could be expanded or are missing in current care? |  | |  |
| **Facilities and Equipment** | **Could you describe where exercise therapy consultations usually take place and what technical tools or resources you use during these sessions?** | - Are there any specific areas or resources where you see a need for improvement? - How satisfied are you with the facilities and equipment available at your institution to deliver and coordinate exercise therapy for patients? | |  | |
| **Special support services** | **Not everyone has the same opportunities to access healthcare services. (language barriers, physical or mental limitations, or social background). Can you tell me about the special support services you offer to ensure that everyone has the best possible access to exercise therapy?** | - - People with disabilities? - - Offers in easy language? - - Services in other languages? - - Single parents: Childcare during sport? - - Offers for women? | |  | |
| **Networks of exercise therapy**  **(intraorganisational)** | **When you think about the collaboration in your exercise unit, which people/professional groups do you work particularly closely with when it comes to providing patients with exercise oncology services?** | - How would you describe the cooperation, communication and general working atmosphere in your team? - Who else would you like to see in the interaction? - Opportunities, challenges, needs? - Roles of hierarchies and functions? | |  | |
| **Interdisciplinary networks**  **(interorganisational)** | **How does collaboration with other professional groups work overall in the CCC?** |  |  |  | |
| **Regional networks** | **With which institutions outside the CCC do you cooperate in the care process?** |  |  |  | |
| **The study's expectations** | **What do you hope to achieve with the MOVE ONKO project?** | - What do you still need before starting the implementation? Where do you see difficulties? - What is your personal motivation for participating in the project? | |  | |
| Is there anything else you would like to pass on to us// is there anything else we haven't talked about yet that you would like to tell us? | | | | | |
| **End and goodbye** | | | | | |

**Supplement IV: Codebook and process of data analysis**

| **Steps of analysis** | **Main categories** | **Sub categories** | **Definition /Application** | **Example** |
| --- | --- | --- | --- | --- |
| **Contextual care settings** | **Current care process** | Need assessment & patient information | When respondents talk about assessing the need for exercise therapy counselling (e.g. in tumour conferences or by questionnaires) and how patients are informed about exercise therapy options. | *So, when the patients have their first appointment here at the centre, our guidance service hands out a tablet to them with a questionnaire. There are questions about quality of life, nutritional value, etc. after which the guidance service contacts the patient if it needs something specific. (4656, item 21)* |
|  |  | Initial contact | When respondents talk about the initial contact in the context of active contact (by the team or the patients) between exercise therapy and patients. | *And then we call and say we need something or you've expressed interest, and then we offer advice on physical exercise. (1568, item 19)*  *Well it varies, but it's always the case that people show up here somehow. They either simply appear at the door and knock (...) or they call. (1715, item 9)* |
|  |  | Counselling & Advice | When respondents talk about the process, potential for improvement and/or resources of exercise therapy counselling and/or patient information. | *While advice on training - with the patients coming to us and getting advice - used to be the situation up until the pandemic, a lot of it is now done via video, with patients from the hospital essentially being made aware of the services offered through its facilities. And they then get this advice here. (2539, item 3)* |
|  |  | Referral into exercise therapy interventions | When respondents talk about the utilisation and/or uptake of internal services and/or external services. | *And then we look to see what's around in their neighborhood, perhaps a physical therapy centre, maybe a rehabilitation centre, and then we call these specifically to see if they have people or therapists who also know something about cancer diagnosis (...). (1568, item 27)* |
|  | **Development of exercise therapy care** | | When respondents talk about the dissemination and generation of knowledge and offers on oncology exercise therapy care and/or about establishing new contacts for progression, for example at conferences, training courses and/or within other networks. | *I think what there is so far depends to an extreme extent on the commitment of individual people, and I think that's a real shame. […] And, as I said, without all that private commitment, none of the locations would be anywhere near where they are now. And, of course, I also hope this project will make things a bit easier, because it’s been a very rocky road here so far, despite a huge amount of backing and support. (4230, item 148)*  *You would imagine a huge hall with 30 or 40 items of equipment. Well, it’s not like that at all. But it is a start. And, as I said, this wasn't even possible a few years ago, but it's been set up very quickly, so we can now offer around three training sessions per week. (1568, item 15)* |
| **Qualitative network analysis** | **Network structures** | Internal actors | Which actors within the institution are involved in the collaboration with access to exercise therapy interventions? Application of the category: When respondents name actors within the institution.  Distinctions: Here, it is sufficient to simply code the named actors. As soon as the content description of a relationship is discussed (the purpose of the collaboration), it should be assigned to a different category, e.g. ‘Network relationships -> internal cooperation/communication’. | **See deepening steps / Results for Principles** |
|  |  | External actors | Which external actors are involved in the collaboration? Application of the category: When respondents name other actors (individuals/institutions/organisations) outside the institution.  Delimitations: Here, it is sufficient to simply code the named actors. As soon as the content description of a relationship is discussed (the purpose of the collaboration), this should be assigned to a different category, e.g. ‘Network relationships -> external cooperation/communication’. |  |
|  |  | Missing actors | Not further analysed. |  |
|  | **Network relationships** | Internal cooperation / communication | How is cooperation (collaboration and communication) carried out within the institution?  Application of the category: When respondents discuss the nature, quality and purpose of cooperation (collaboration and/or communication) within the institution. |  |
|  |  | external cooperation / communication | How is cooperation (collaboration and communication) carried out outside the institution?  Application of the category: When respondents talk about the nature, quality and purpose of cooperation (collaboration and/or communication) with other actors (external persons or health care facilities (rehabilitation clinics, physiotherapy practices, registered oncology practices, etc.) or the implementation of recommendations by external partners. |  |
| **Deepening Steps of Network relationships analysis based on subcategories: internal and external cooperation / communication and internal and external actors** | | | | |
| **Steps of analysis** | **Steps** | | **Definitions** | **Example** |
| **Generating: principles of operation** | 1. Wo is available: Which copresence is stated? And direction of the cooperation | | Extraction of the coded actors in one stated cooperations in the interviews. | Exercise Therapy <-> Psycho Oncology  (bilateral/unilateral) |
|  | 1. Why is this copresence present? | | The purpose of the copresence when it comes to cooperation was paraphrased and categorised into further subcategories. | - Knowledge about exercise therapy care - Interdisciplinary care - Development of exercise therapy care |
|  | 1. What overarching pattern in networks are presented? | | Relevant overarching pattern of principles of operation are derived from step two. | See results: Figure 2: Principles of operation |

**Supplement V: Principles of Operation per CCC**

| **Network structures (actors and relationships)** |  | **Site / CCC** | | | | | | | **Definition Principals** |
| --- | --- | --- | --- | --- | --- | --- | --- | --- | --- |
|  | **Principles** | **1** | **2** | **3** | **4** | **5** | **6** | **7** |  |
| **Supportive Services / Tumour Boards** | *Reciprocity* |  | ***X*** | ***X*** | ***X*** |  | ***X*** | ***X*** | Through already implemented joint case discussions, or even specialised tumour boards, resources can be pooled in a targeted manner and joint care agreements can be made. In addition to the social capital that is generated, the principle of reciprocal mutuality usually comes into play, enabling disciplines to coordinate and to communicate information to patients across professional boundaries. |
| **Patient Navigators** | *Sensibilisation and Awareness* | ***X*** |  | ***X*** | ***X*** | ***X*** | ***X*** | ***X*** | Through patient navigators who are already in place, pathways within clinics can be facilitated and made more accessible. In particular, exercise therapy is often presented merely as a separate room or training area, meaning that it is not immediately obvious to patients where it can be found. In some cases, the navigators are already involved in exercise therapy and its screening, thereby enabling comprehensive and holistic care. |
| **(Structured) Screening** | *Needs Planning* | ***X*** |  | ***X*** | ***X*** |  | ***X*** | ***X*** | Through already implemented a structured screening for some or all supportive services at the start of treatment, patient care can potentially be better tailored to individual needs. Crucial factors include the timing and repetition of such screenings, as well as the proper processing of the data generated. |
| **Medical Care** | *Collaboration* | ***X*** | ***X*** | ***X*** | ***X*** | ***X*** | ***X*** | ***X*** | Through close collaboration with sports medicine or other medical care disciplines, or when exercise therapy has even originated from sports medicine, a cooperation between the disciplines is established. It becomes clear that medical input as a consultative service is indispensable for the implementation of exercise therapy interventions. The closer the medical team is to exercise therapy care team, the more easily consultations across all specialties can be obtained. This is further facilitated when individual physicians emphasise and advocate the importance of exercise therapy interventions, and when a tandem of exercise specialists and physicians is present during patient consultations. |
| **Informational Events** | *Knowledge Building* | ***X*** |  | ***X*** | ***X*** |  |  | ***X*** | Through informational events and small internal training sessions, the importance of primary care providers within the exercise therapy care system is repeatedly emphasised. Ongoing staff turnover and high fluctuation present obstacles, making continuous events necessary. Nevertheless, building knowledge among primary care providers represents an effective strength within the system. |
| **Recent Projects / Joint Studies and Structures** | *Mutual Recognition* |  | ***X*** | ***X*** | ***X*** | ***X*** | ***X*** | ***X*** | Through shared structures from previous projects, the disciplines have gained deeper knowledge of each other, which can be leveraged as social capital at the organisational level in new projects. This significantly facilitates the development of future structures and collaborative working practices. |
| **Exercise Therapy** | *Accessibility* |  |  | ***X*** | ***X*** | ***X*** | ***X*** | ***X*** | Through existing on-site exercise programmes, patients receive support, and participants recruit and motivate each other, creating a snowball effect and accessibility is easily gained for patients. |

1. Day treatments [↑](#footnote-ref-1)
2. UCT: University Center for Tumor Diseases [↑](#footnote-ref-2)
3. Own calculation: based on Case-Mix: 178.307 [↑](#footnote-ref-3)
4. ITZ: Interdisciplinary Tumor Center, an outpatient center that serves as a central point of contact for patients. [↑](#footnote-ref-4)
